# Anti-Inflammatory and Physicochemical Characterization of the *Croton rhamnifolioides* Essential Oil Inclusion Complex in β-Cyclodextrin

**DOI:** 10.3390/biology9060114

**Published:** 2020-05-30

**Authors:** Anita Oliveira Brito Pereira Bezerra Martins, Almir Gonçalves Wanderley, Isabel Sousa Alcântara, Lindaiane Bezerra Rodrigues, Francisco Rafael Alves Santana Cesário, Maria Rayane Correia de Oliveira, Fyama Ferreira e Castro, Thaís Rodrigues de Albuquerque, Maria Sanadia Alexandre da Silva, Jaime Ribeiro-Filho, Henrique Douglas Melo Coutinho, Paula Passos Menezes, Lucindo José Quintans-Júnior, Adriano Antunes de Souza Araújo, Marcello Iriti, Jackson Roberto Guedes da Silva Almeida, Irwin Rose Alencar de Menezes

**Affiliations:** 1 Laboratory of Pharmacology and Preclinical Bioactive Product Toxicology Federal University of Pernambuco, Av. Prof. Moraes Rego, 1235—Cidade Universitária, Recife 50670-901, Pernambuco, Brazil; anitaoliveira24@yahoo.com.br (A.O.B.P.B.M.); almirgw.wanderley@gmail.com (A.G.W.); 2 Laboratory of Pharmacology and Molecular Chemistry, Department of Biological Chemistry, Regional University of Cariri, Rua Coronel Antônio Luis 1161, Pimenta, Crato 63105-000, Ceará, Brazil; isabel-alcantara-@hotmail.com (I.S.A.); lindaianebrd@gmail.com (L.B.R.); rayaneoliveirabio@gmail.com (M.R.C.d.O.); fyama_fc@hotmail.com (F.F.e.C.); thaysrodrigues_albuquerque@hotmail.com (T.R.d.A.); sanadiaalexandre@gmail.com (M.S.A.d.S.); irwinalencar@yahoo.com.br (I.R.A.d.M.); 3 Laboratory of Pharmacology and Molecular Chemistry, Department of Physiology and Pharmacology, Federal University of Ceara, Rua Coronel Nunes de Melo, 1127—Rodolfo Teófilo, Fortaleza 60430-275, Ceará, Brazil; rafa.san@hotmail.com; 4 Gonçalo Moniz Institute, Oswaldo Cruz Foundation (IGM-FIOCRUZ/BA), Rua Waldemar Falcão, 121, Candeal, Salvador 40296-710, Bahia, Brazil; jaimeribeirofilho@gmail.com; 5 Laboratory of Microbiology and Molecular Biology, Department of Biological Chemistry, University of Cariri; Rua Coronel Antônio Luis 1161, Pimenta, Crato 63105-000, Ceará, Brazil; 6 Laboratory of Pharmaceutical Testing and Toxicity, Department of Pharmacy, Federal University of Sergipe; Avenida Marechal Rondon, S/N, São Cristóvão 49100-000, Sergipe, Brazil; paulamenezes_16@yahoo.com.br; 7 Laboratory of Neuroscience and Pharmacological Assays, Department of Physiology, Federal University of Sergipe; Avenue Marechal Rondon, S/N, São Cristóvão 49100-000, Sergipe, Brazil; lucindo_jr@yahoo.com.br (L.J.Q.-J.); adriasa2001@yahoo.com.br (A.A.d.S.A.); 8 Department of Agricultural and Environmental Sciences, Milan State University, via G. Celoria 2, 20133 Milan, Italy; marcello.iriti@unimi.it; 9 Laboratory of Pharmacology, Department of Pharmacy, Federal University of Vale Sao Francisco, Av. José de Sá Maniçoba, s/n, Campus Petrolina Centro, Petrolina 56304-205, Pernambuco, Brazil; jackson.guedes@univasf.edu.br

**Keywords:** *Croton rhamnifolioides*, cyclodextrin, inflammation

## Abstract

*Croton rhamnifolioides* is used in popular medicine for the treatment of inflammatory diseases. The objective of this study was to characterize and evaluate the anti-inflammatory effect of *C. rhamnifolioides* essential oil complexed in β-cyclodextrin (COEFC). The physicochemical characterization of the complexes was performed using different physical methods. The anti-inflammatory activity was evaluated in vivo by ear edema, paw edema, cotton pellet-induced granuloma, and vascular permeability by Evans blue extravasation. The mechanism of action was validated by molecular docking of the major constituent into the cyclooxygenase-2 (COX-2 enzyme). All doses of the COEFC reduced acute paw edema induced by carrageenan and dextran, as well as vascular permeability. Our results suggest the lowest effective dose of all samples inhibited the response induced by histamine or arachidonic acid as well as the granuloma formation. The complexation process showed that the pharmacological effects were maintained, however, showing similar results using much lower doses. The results demonstrated an involvement of the inhibition of pathways dependent on eicosanoids and histamine. Complexation of β-cyclodextrin/Essential oil (β-CD/EO) may present an important tool in the study of new compounds for the development of anti-inflammatory drugs.

## 1. Introduction

Essential oils are complex mixtures of natural compounds obtained from aromatic plants. Because of their volatility, these compounds are characterized by an intense odor [1] and have specific physicochemical characteristics, including liquid composition at room temperature, oily aspect, colorless or slightly yellowish appearance, and low stability when exposed to air, humidity, light, heat, and metals [2]. Thus, many medicinal plants rich in essential oils have their therapeutic effects attributed to the presence of these volatile mixtures. On the other hand, it has been shown that, depending on the chemical composition, essential oils are relatively safe when used in low concentrations; however, they can show signs of toxicity to humans when administered in high concentrations or when administered in repeated doses [3]. The mechanisms involved in the toxicity of these compounds include changes in the permeability of the cell membrane and the mitochondrial membrane, causing cell death by necrosis or apoptosis [4]. The complexation in cyclodextrin is an important strategy of the drug delivery system. These complexations can promote better bioavailability [5,6] with the reduction of the effective dose and, consequently, low collateral effects [7]. 

Terpenes, especially monoterpenes and sesquiterpenes, are the most abundant substances found in essential oils [1]. The chemical diversity of the essential oils is influenced by genetic and seasonal factors that alter the composition of these mixtures. However, despite these influences, major compounds can reach up to 85% of the oil composition [8,9]. Monoterpenes have appreciable biological properties, such as analgesic, anti-inflammatory, cicatrizing, neuroprotective, antioxidant, and antitumor, among other properties, and therefore, are compounds of interest to the pharmaceutical and cosmetic industries [10,11,12]. Studies have demonstrated that the anti-inflammatory potential of monoterpenes is associated with a wide range of mechanisms, such as the inhibition of enzymes responsible for the inflammatory response (including cyclooxygenase-2 (COX-2), inducible nitric oxide synthase (iNOS), and lipoxygenase), inhibition of the activity of lymphocytes and natural killer cells, inhibition of the production of proinflammatory cytokines (such as: interleukin-1β(IL-1β), interleukin-6 (IL-6), and alpha tumor necrosis factors TNF-α) and modulation of translocation of transcriptional factors, such as nuclear factor kappa B (NF-κB) [13,14]. Regarding the toxicity of these compounds, Vigan (2010) [15] reported that limonene ingestion caused diarrhea and transient proteinuria in healthy volunteers [15]. In addition, *cis*-thujone has been shown to be neurotoxic and implicated in the effects of absinth consumption [16].

The monoterpene 1,8-cineole (synonym: eucalyptol) is a constituent of essential oils of many plant species, such as *Croton pulegioides* [17] and *Cannabis sativa* [18]. Previous reports demonstrated that this compound is biologically active, presenting anti-inflammatory, antinociceptive [16,17], gastroprotective [19], antioxidant [20], anti-hypertensive [21], antifungal [22], and antibacterial [23] activities. However, like other monoterpenes, 1,8-cineole has low solubility in water, which may limit its therapeutic applications [24]. Additionally, when administered at high concentrations or by repeated doses, this monoterpene caused alterations in hematological and biochemical parameters, in addition to histological changes in the lungs, liver, kidneys, and uterus [25].

*Croton rhamnifolioides* PAX. & K. HOFFM (Euphorniaceae), popularly known as “quebra-faca”, is a plant species used in folk medicine to treat stomach pain, sinusitis, headache, vomiting, fever, and bloody diarrhea [26,27,28]. In a recent study, we demonstrated that 1,8-cineol was found as one of the major constituents (41.33%) of the essential oil of *C. rhamnifolioides* and that both the essential oil and 1,8-cineole had anti-inflammatory effects in models of acute and chronic inflammation [29]. However, the chemical fingerprint of OEFC (*Croton rhamnifolioides* leaf essential oil) demonstrates the presence of other terpenes, such as spathulenol (7.89%), caryophyllene oxide (2.84%), *trans*-caryophyllene (5.86%), β-elemene (8.59%), α-terpineol (2.35%), Terpinen-4-ol (2.35%), and α-Phellandrene (4.45%). Previously published studies have also shown that these compounds have anti-inflammatory activity as spathulenol [30], caryophyllene oxide [31], *trans*-caryophyllene [32], β-elemene [33], α-terpineol [34], Terpinen-4-ol [35], and α-Phellandrene [36].

Despite the promising results, the physicochemical characteristics and the risk of toxicity represent challenges for the development of drugs from the oil of *C. rhamnifolioides*. In this context, our group previously demonstrated that the complexation of essential oils or isolated components in cyclodextrins (CDs) increased the bioavailability and reduced the effective dose of these substances [37].

In fact, CDs have unique physicochemical properties, which allow them to solubilize in an aqueous media while encapsulating hydrophobic molecules within its cavity [38]. Thus, as transport systems, they can be used to improve the bioavailability and water solubility, and prevent oxidation, luminosity, heat-induced degradation, processing, and storing loss, as well as promoting the stabilization of aromas and flavors against the oxidative process [39]. Therefore, CDs represent an important alternative for the development of pharmaceutical formulations, including those containing compounds with analgesic and anti-inflammatory properties [24,40,41].

The objective of this study was to perform a detailed physicochemical characterization and evaluate the anti-inflammatory activity of the inclusion complexes of the *Croton rhamnifolioides* essential oil in β-cyclodextrin.

## 2. Materials and Methods

### 2.1. Chemicals

β-Cyclodextrin, arachidonic acid, histamine, carrageenan, *croton* oil, dextran, *Evans* blue, indomethacin, and dexamethasone were purchased from Sigma-Aldrich Corporation (St. Louis, MO, USA), and xylazine and ketamine (Ceva SantéAnimale, São Paulo, Brazil).

### 2.2. Extraction of the Croton Rhamnifolioides Essential Oil

The leaves of *C. rhamnifolioides* were collected at the Riacho da Caatingueira site (Aiuaba-CE, Brazil) between May and June 2014 (Authorization-ICMBio No. 47705-1). A representative sample was identified by Prof. Dr. Maria Arlene Pessoa da Silva and deposited in the Caririense Dárdano de Andrade Lima Herbarium of the Regional University of Cariri (exsiccate No. 12.062). The essential oil was extracted from fresh leaves using a hydrodistillation system in a Clevenger-type apparatus. In total, 11 constituents were identified, with 1,8-cineole as the main component. The chemical composition of the essential oil was performed by GC-MS using a Shimadzu QP-2010 gas chromatographic coupled to a mass spectrometer (GC-MS) was described in our recently published paper [29]. 

### 2.3. Preparation of Inclusion Complexes

Inclusion complexes were prepared at the Pharmaceutical Testing and Toxicity Laboratory of the Federal University of Sergipe, using the following techniques: (1) Physical mixture (MF): The OEFC (154.25 mg, based on the molecular weight of the OEFC major constituent, 1,8-cineole), and β-CD (1135.00 mg) were mechanically mixed at a 1:1 molar ratio for 40 min by a magnetic stirring device operating at 400 rpm under ambient conditions; (2) Malaxage (MA): The OEFC (154.25 mg) and β-CD (1135.00 mg) were mixed (1:1 molar ratio), then 1 mL of distilled water was added and the preparation was homogenized manually with a mortar and pestle for 40 min; and (3) co-evaporation (CE): The OEFC (771.25 mg) and β-CD (5675.00 mg) were mechanically mixed in a 1:1 molar ratio (5X) in 20 mL of distilled water for 40 min by a magnetic stirring device operating at 400 rpm under constant stirring for 36 h/240 rpm and subsequently dried in a glass desiccator with silica [42].

### 2.4. Physicochemical Characterization of the Inclusion Complexes

The differential scanning calorimetry (DSC) curves (OEFC, β-CD, MF, MA, and CE) were obtained using a DSC-50 cell (Shimadzu, Kyoto, Japan), at a heating rate of 10 °C/min, at 25–500 °C, under an N_2_ dynamic atmosphere (50 mL/min). An aluminum (Al) capsule containing approximately 2 mg of the sample was used in this assay. For the TG/DTG (thermogravimetry/derivative thermogravimetry) assays, a TGA-51 thermogravimetric analyzer (Shimadzu, Kyoto, Japan) was used at a temperature range of 25–500 °C, under a dynamic N_2_ atmosphere (50 mL/min). A platinum crucible (Pt) containing approximately 2 mg of the sample was used in this assay. For the MeV analysis, the samples were mounted in aluminum tubes, metallized with gold beams, and visualized in an electronic microscope (model JSM-6390-LV; JEOL, Peabody, MA, USA) under a voltage acceleration of 12 kV. The moisture content (OEFC, β-CD, MF, MA, and CE) was determined using the Karl Fischer method with the aid of a Titrino Plus KF 870 (Metrohm, Herisau, Switzerland), using methanol (Fluka, St. Louis, MO, USA) as the titration solution. The analyses were performed in triplicates [42].

### 2.5. In Vivo Experimental Protocols

The inclusion complex selected for the in vivo protocols was obtained by the technique that demonstrated the best complexation (co-evaporation). The doses used in the oral treatments were 8.35, 41.75, and 83.5 mg/kg and the topically administered concentrations were 0.83, 4.17, and 8.35 mg/mL. For OEFC and 1,8-cineole, doses were determined in the previous protocols of Martins et al. (2017) [29]. The results of *Croton rhamnifolioides* leaf essential oil (OEFC) and 1,8-cineole were obtained from our previously published research [29] and included in this paper, with the objective of comparing the effects of the complexed and uncomplexed essential oil. Prior to the administration, the treatment solutions were prepared to obtain a 0.1 mL/10 g body mass proportion, according to the specific protocol. The doses less than 5% of the LD_50_ of *Croton rhamnifolioides* essential leaf oil complexed in β-cyclodextrin (COEFC) were used in the protocols assay.

### 2.6. Animals

Male Swiss mice (Mus musculus) weighing 25 ± 5 g were used in the experiments. The animals were housed in polypropylene cages and maintained at a temperature of 23 ± 2 °C, in a 12-h light/dark cycle, with free access to potable water and rodent-specific ration. However, they were fasted of solid food 8–10 h before the tests. The animals were euthanized in a CO_2_ box. The experimental protocols were submitted and approved by the Animal Research Ethics Committee of the Regional University of Cariri (CEUA/URCA No. 43/2015.1) in accordance with the National Institute of Health’s (Washington, DC, USA, 2011) Guide for the Care and Use of Laboratory Animals.

### 2.7. Determination of the Median Lethal Dose (LD_50_)

The animals (*n* = 3) were treated with a single oral dose of COEFC (2000 mg/kg) or H_2_O (control group). Following treatment, the animals were observed at the following time periods: 30 min, 60 min, 240 min, 360 min, 24 h, and daily for 14 days. The LD_50_ was determined from the number of deaths according to OECD 425 [43,44,45].

### 2.8. Ear Edema Induced by a Single Administration of Croton Oil

This protocol was carried out according to the methodology described by Tubaro et al. (1986) with few modifications [46] as follows: The animals (*n* = 6) received a topical treatment in the right ear (10 μL on the inner face of the ear + 10 μL on the outer face of the ear) with H_2_O (control), dexamethasone (4 mg/mL) and COEFC (0.83, 4.17, and 8.35 mg/mL) diluted in water and Tween 80. After 60 min, 20 μL of 5% (*v*/*v*) *croton* oil in acetone were applied in the right ear (OD), and 20 μL of the acetone control in the left ear (OE). After 6 h, the animals were euthanized, and 6-mm-diameter discs were removed from each ear with the aid of a punch (metallic leather punch) to evaluate the inflammation percentage, as follows: Inflammation percentage = (MOD − MOE-being/MOE-being) × 100, in which MOD and MOE-being: masses (g) of the discs of the right and left ears, respectively. Percent inhibition (%) was calculated as: 100%—inflammation percentage.

### 2.9. Paw Edema Induced by an Intraplantar Administration of Carrageenan

In the carrageenan-induced paw edema model, the animals (*n* = 6) were divided into groups and treated as follows: Control (saline, p.o.); indomethacin (10 mg/kg, s.c.); and COEFC (8.35, 41.75, or 83.5 mg/kg, p.o.) diluted in water and Tween 80. After 30 or 60 min, the animals received 1% (*w*/*v*) carrageenan (20 μL/paw) in the right hind paw and 0.9% saline (20 μL/paw) in the left paw. The volumes of the right and left hind paws of each animal were recorded 60, 120, 180, and 240 min after carrageenan administration [47].

### 2.10. Paw Edema Induced by an Intraplantar Administration of 1% Dextran

For the evaluation of dextran-induced paw edema, the animals (*n* = 6) were divided into groups and treated as follows: Control (saline, p.o.); promethazine (6 mg/kg, p.o.); and COEFC (8.35, 41.75, or 83.5 mg/kg, p.o.) diluted in water and Tween 80. After 60 and 30 min of the oral and subcutaneous treatments, respectively, the animals received the inducing agent (1% dextran (*w*/*v*)) in the right hind paw (20 μL/paw) and 0.9% saline in the left hind paw (20 μL/paw). The right and left hind paw volumes of each animal were recorded 60, 120, 180, and 240 min after dextran administration [47].

### 2.11. Paw Edema Induced by an Intraplantar Administration of Histamine

This test was performed according to the model described by Maling et al. (1974) and de Oliveira Ramalho (2015) [48,49]. Briefly, the animals (*n* = 6/mice) were divided into groups and pre-treated as follows: Control (saline, p.o.); promethazine (6 mg/kg, p.o.); and COEFC (8.35 mg/kg, p.o.) diluted in water and Tween 80 [48,49]. After 60 min, the animals received 1% histamine (*w*/*v*) in the right hind paw (20 μL/paw) and 0.9% saline (20 μL/paw) in the left hind paw. The volumes of the right and left hind paws of each animal were recorded 30, 60, 90, 120, and 180 min after histamine injection.

### 2.12. Paw Edema Induced by an Intraplantar Administration of Arachidonic Acid

The animals (*n* = 6) were divided into the following treatment groups: Control (saline/tween 80 (10 mg/kg), p.o.); indomethacin (10 mg/kg, s.c.); and COEFC (8.35 mg/kg, p.o.) diluted in saline and Tween 80. Subsequently, 60 and 30 min after the oral and subcutaneous treatments, respectively, the animals received 1% arachidonic acid (*w/v*, 20 μL/paw) in the right hind paw and vehicle (20 μL/paw) in the left hind paw. The volume of the right and left hind paws from each animal were recorded 15, 30, 45, 60, and 90 min after the administration of arachidonic acid [50,51].

### 2.13. Paw Edema Measurement

In all experimental models, paw edema was analyzed by plethysmometry. The animals had the initial volume (Vi) of their right and left hind paws evaluated before the treatments. Then, differences between the final volume and the basal volume of the paws at each time was calculated using the formula: Ve.p.d/e = Vf − Vb, in which Ve.p.d/e = right/left paw edema volume (in μL), Vf = final volume of the right and left paw/time, and Vb = basal volume of the right and left paw/time. Afterwards, the Δ (delta) of the following formula was calculated: Δ = Ve.p.d − Ve.p.e. The percentage of inflammation between treated and control groups was calculated using the following formula: Inflammation (%): 100 × Δmt/Δmc, in which Δ mc and Δ mt represent the mean paw volume in the control and treated groups, respectively. Percent inhibition was calculated as: 100%—percentage of inflammation.

### 2.14. Evaluation of Vascular Permeability by Evans Blue Extravasation

The animals (*n* = 6/mice) were treated according to the following groups: Control (saline, p.o.); baseline control (no inducing agent); indomethacin (10 mg/kg, s.c.); and COEFC (8.35 mg/kg, p.o.) diluted in water and Tween 80. Shortly after the treatments, 1% Evans Blue (0.2 mL/animal) was administered in the retro orbital plexus. Then, 60 and 30 min after oral and subcutaneous treatments, respectively, 1 mL of 1% carrageenan (i.p.) was injected. After 4 h of induction, the animals were euthanized by cervical dislocation and injected with 3 mL of PBS (phosphate buffered saline) into the peritoneum. A peritoneal massage was performed, followed by asepsis with 70% alcohol, collection of the washed biological material (1.5 to 2 mL), and centrifugation (6000 rpm/3300 g/2 min/20 °C). Subsequently, protein dosage was performed as previously described [52]. In order to determine the total level of proteins, the enzymatic method using the Labtest (Minas Gerais, Brazil) kits were used, in which copper ions (Cu2+) in an alkaline medium (biuret reagent) react with the peptide bonds from serum proteins, forming a purple staining whose absorbance, measured at 545 nm (by ELISA), is directly proportional to the protein concentration of the sample.

### 2.15. Granuloma Induced by Cotton Pellet Implantation

Animals (*n* = 6/group) previously anesthetized with ketamine (80 mg/kg/i.p.) and xylazine (20 mg/kg/i.p.) had 4 cotton pellets weighing approximately 10 mg (0.01 g) implanted in their back by means of a small dorsal incision. The animals were treated for 10 days as follows: Control (saline, p. o.), dexamethasone (5 mg/kg, p.o.), and COEFC (8.35 mg/kg, p.o.). After this period, the animals were euthanized, the cotton pellets were removed, as well as the surrounding fibrovascular tissue, and this material was weighed, dried in an incubator (40 °C /24 h), and weighed again. The results were expressed as the difference between the initial and final dry mass of the pellets [53]. At the end of this process, the total proteins present in the pellet homogenates were quantified.

### 2.16. Statistical Analysis

The results were expressed as mean ± standard error of the mean (S.E.M). Differences between groups were analyzed by one-way and/or two-way analysis of variance (ANOVA) using Dunnett’s multiple-comparison tests. The results were analyzed using the GraphPad Prism Version 7.0 statistical software (GraphPad Software, Inc. La Jolla, CA, USA) and *p* < 0.05 was considered as significant.

### 2.17. Docking Procedure and Pharmacokinetic Characteristics

The X-ray crystal structure of cyclooxygenase-2 (COX-2) from Mus musculus (PDB ID: 1PXX) was retrieved from a protein data bank (www.pdb.org). Then, the water molecules were removed, and the enzymes cleaned from any unwanted interactions. To ensure the correct ionization and tautomeric amino acid residue states, all nonpolar hydrogens were fused (removed) and partial atomic charges were assigned using the CHARMM force field. Then, charges were added to enzyme structures and the CHARMM force field and atomic salvation parameters were assigned. Moving forwards, incomplete side chains were replaced using the Dunbrack rotamer library using the Chimera 1.8 software. Molecular docking was carried out in order to evaluate a possible binding mode between the COX-2 receptor and 1,8-cineole, prostaglandin E2 natural ligand, diclofenac (non-selective COX-1/COX-2) inhibitor), naproxen and meloxicam (moderately selective COX-2 inhibitors), and celecoxib (selective COX-2 inhibitor). Docking studies were performed using the online SwissDock server (http://www.swissdock.ch/docking) [54]. 

## 3. Results

### 3.1. Physico-Chemical Characterization of the OEFC Inclusion Complex in β-Cyclodextrin

**Figure 1:**
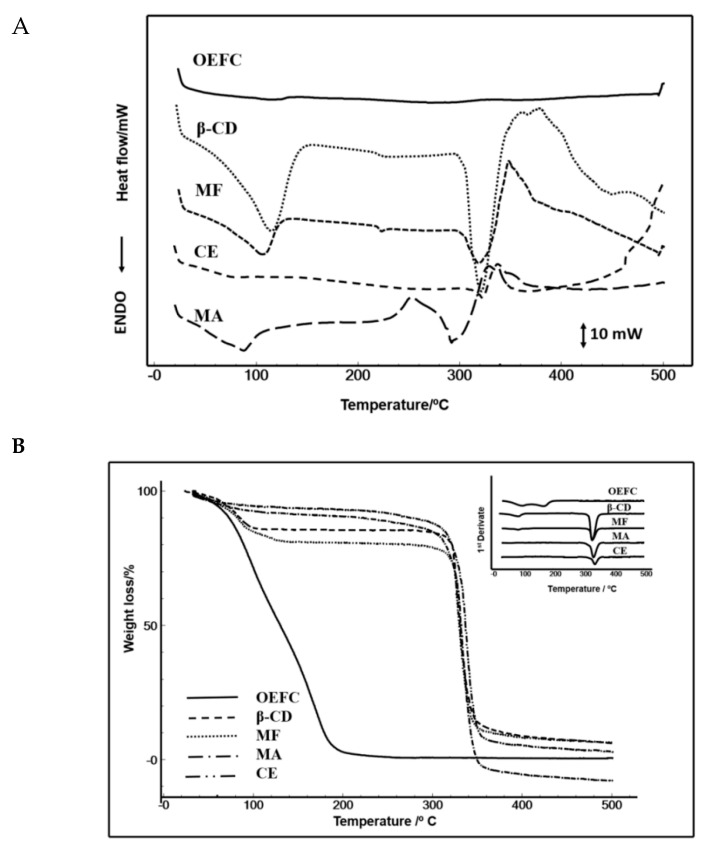
Differential scanning calorimetry (DSC) (**A**) and thermogravimetry (TG) (**B**) of β cyclodextrin (β-CD) obtained by physical mixture (MF), malaxation (MA), and co-evaporation (CE), in a nitrogen inert atmosphere. Curves from the complex *Croton rhamnifolioides* essential oil (OEFC).

In Figure 1A, the OEFC differential scanning calorimetry (DSC) showed an endothermic event in the temperature range of 30–141 °C (enthalpy= −139.13 mJ) characterized by the rapid volatilization of the essential oil. The β-CD DSC curve presented three events, followed by a decomposition stage. The first endothermic event occurred in the 30–156 °C temperature range (enthalpy = 1.40 mJ) associated with dehydration of the molecule. The second event occurred in the temperature range of 213–240 °C (enthalpy = −11.03 mJ) characteristic of the crystalline phase transition. The third event occurred in the temperature range of 298–360 °C (enthalpy = −1.17 mJ) characterized by β-CD fusion followed by β-CD degradation.

The DSC curve of the physical mixture (MF) (Figure 1A) presented three endothermic events followed by decomposition. The first event represented the sum of the OEFC and β-CD, characterized by dehydration in the temperature range of 29–136 °C (enthalpy = −546.68 mJ). The second event, characterized by phase transition, occurred in the temperature range of 217–230 °C (enthalpy = −7.19 mJ), demonstrating that there was no complexation since the same event occurred with β-CD. The third event occurred in the temperature range of 297–349 °C (enthalpy = −573.8 mJ), characteristic of β-CD fusion followed by decomposition.

The malaxage (MA) DSC curve (Figure 1A) presented two endothermic events followed by decomposition. The first event occurred in the temperature range of 30–128 °C (enthalpy = −110.06 mJ), indicating that dehydration occurred without the formation of an effective complex. However, this occurred in a lower proportion than for β-CD and MF. The second event occurred in the temperature range of 306–339 °C (enthalpy = −84.81 mJ) characterized by fusion of β-CD followed by decomposition. The co-evaporation (CE) DSC curve (Figure 1A) did not show endothermic events, which justify a complexation through OEFC coupling in the β-CD cavity followed only by decomposition in the temperature range of 252–324 °C (enthalpy = −684.96 mJ), characterized by β-CD fusion.

Figure 1B and Table 1 represent the thermogravimetry/derivative thermogravimetry (TG/DTG) curves, in which the OEFC lost mass up to 220 °C, as follows: 97.20% in the first interval (30–220 °C) and 2.3% at the 2° interval (220–270 °C). β-CD lost mass in four stages: In the first temperature interval (30–220 °C), a 12.8% mass loss was observed, attributed to water release from the β-CD structure. In the interval of 220–270 °C, no significant mass loss (0.1%) was observed, characterizing the transition phase previously described in the DSC curve. The temperature interval of 270–380 °C indicated the beginning of the β-CD decomposition process with a mass loss of 76.6%. In the interval of 380–500 °C, a mass loss of 3.7% was observed.

In Table 1, the MF, MA, and CE samples had 19.4%, 10.1%, and 8.5% of the mass losses in the first interval (30–220 °C), respectively. In the second interval of temperature (220–270 °C), the samples showed the following mass loss percentages, respectively: 0.5%, 2.4%, and 2.6%. In the third temperature range (270–380 °C), the samples presented the following losses, respectively: 70.9%, 92.2%, and 83.4%. In the fourth temperature range, the following mass losses occurred, respectively: 2.7%, 3.2%, and 3.2%. In view of these events, the complexation of the OEFC with the β-CD cavity was most effective in the MA and CE methods, because they required greater temperatures to lose mass when compared to the OEFC. Therefore, the smaller the mass loss, the better the complexation.

In Table 1, the Karl Fisher results corroborate with the TG/DTG curves (Table 1). With regards to the OEFC, a 0.91 ± 0.06% water content was observed, demonstrating an efficient essential oil extraction. In addition, the β-CD water content (13.75 ± 0.39%), was similar to others described in the literature [42,55]. However, the water content of the physical mixture (MF), malaxage (MA), and co-evaporation (CE) methods were 13.45 ± 0.78%, 11.27 ± 0.32%, and 12.80 ± 0.27%, respectively. These results demonstrate that the physical mixture was unable to form inclusion complexes with the OEFC. However, with the malaxation and co-evaporation methods, a reduction in the water content was observed, which in turn suggests complexation due to the substitution of water molecules in the β-CD cavity by OEFC molecules, according to Hădărugă, Hădărugă, and Isengard (2012) [55].

**Figure 2:**
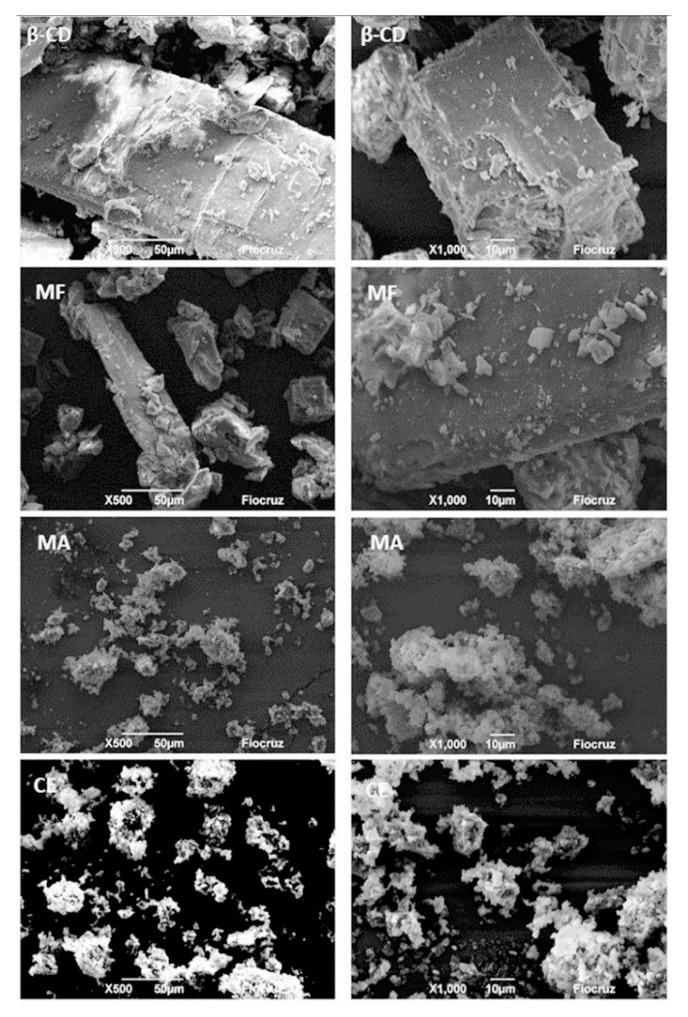
Scanning electron microscopy with magnifications of 50 µm (**left**) and 10 µm (**right**) obtained at 12 kV voltage acceleration. β cyclodextrin (β-CD), physical mixture (MF), malaxation (MA), and co-evaporation (CE).

Figure 2 shows the microphotographs obtained by scanning electron microscopy (MeV) with two different magnitudes of 50 µm (left) and 10 µm (right), in which well-defined β-CD rectangular crystals were observed, as previously described in other studies [55,56]. In the molecular inclusion methods, the physical mixture presents morphological similarities to β-CD, indicating that this is not such an efficient method to form inclusion complexes with EOFC and β-CD, as already demonstrated by the analytical techniques. However, the malaxation and co-evaporation methods showed a dysphormic morphology and a reduction in particle size, as well as aggregate formation with an undefined morphology. This behavior reveals the complexation of substances in cyclodextrins [57]. These results corroborate with the other techniques: DSC, DTG, and Karl Fischer, pointing to the complexation of the OEFC in the β-CD cavity by these methods.

### 3.2. Determination of the Median Lethal Dose (LD_50_)

During the experimental period, the animals were examined daily for the clinical aspects. However, there was no evidence of behavioral changes, no significant physical changes, and no deaths in the experimental groups were observed. Additionally, there were no significant gains in weight, water, and food intake. Therefore, the median lethal dose (LD_50_) is greater than 2000 mg/kg.

### 3.3. Ear Edema Induced by a Single Application of Croton Oil

**Figure 3:**
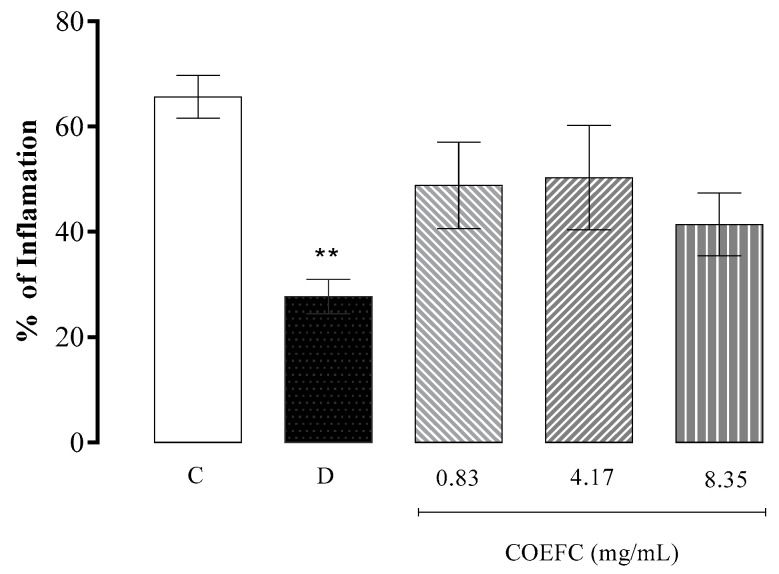
Effect of the topically applied COEFC–*C. rhamnifolioides* essential oil complexed in β-Cyclodextrin (0.83, 4.17, and 8.35 mg/mL) on ear edema induced by a single *croton* oil application in mice. Results of the mean value of the edema percentage between the control (C), dexamethasone (D) (4 mg/mL), and COEFC (0.83, 4.17, and 8.35 mg/mL) groups. Values represent the mean ±E.P.M. (standard error of the mean) for groups of 6 animals. Being, ** *p* < 0.01; vs. control; Statistical analysis: ANOVA followed by the Dunnett’s test with multiple comparisons.

In Figure 3, it is demonstrated that a topical treatment with the COEFC at the concentrations of 0.83, 4.17, and 8.35 mg/mL did not produce anti-edematogenic effects in comparison with the control group. Moreover, no significant differences (*p* < 0.05) in inflammation percentages were observed, although a tendency of an anti-inflammatory action when comparing the control with the treated groups is suggested. Dexamethasone (4 mg/mL), a steroidal anti-inflammatory drug, used as a positive control, produced a significant edema reduction of 57.86% in comparison with the control group. The OEFC (20 mg/mL) and 1,8-cineole (8.26 mg/mL) produced a significant reduction of 42.1% and 35%, respectively, as demonstrated in our previously published research [29]. This result may be due a possible synergism with other constituents present in the OEFC. In addition, the lipophilic character of the OEFC favors its dermal penetration.

### 3.4. Paw Edema Induced by an Intraplantar Administration of 1% Carrageenan and Dextran 1%

**Figure 4:**
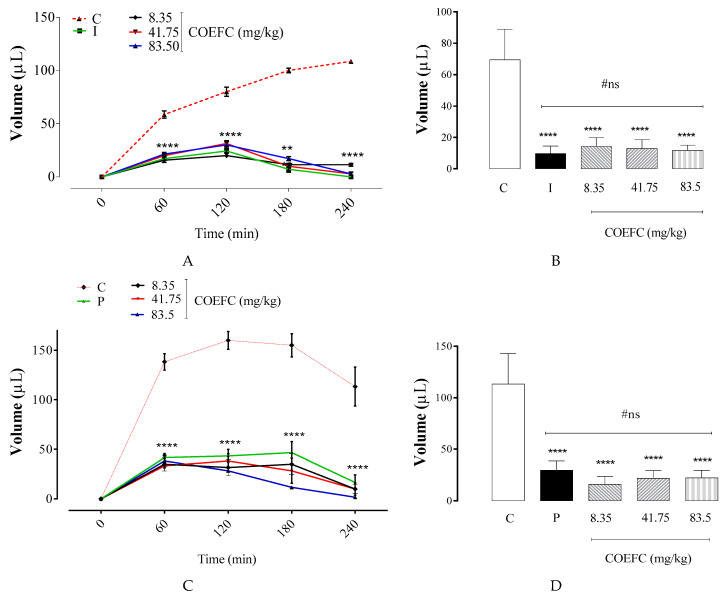
Effect of the COEFC (8.35, 41.75, and 83.5 mg/kg/p.o.) on paw edema induced by the intraplantar injection of 1% carrageenan and 1% dextran in mice. Paw edema induced by the intraplantar injection: (**A**) Edema value (μL) induced by carrageenan and (**C**) edema value (μL) induced by dextran 1%. (**B**,**D**) Results of the median paw volume (μL) at the 4th hour after the moment of application. Evaluation up to the 4th hour between the control groups (C) (saline 0.1 mL/10 g/v.o.), indomethacin (I) (10 mg/kg/s.c.), and COEFC (8.35, 41.75 and 83.5 mg/kg/p.o.). Values represent the mean ± S.P.M. (*n* = 6/group). **** *p* < 0.0001 vs. control; Statistical analysis: ANOVA followed by the Dunnett’s test with multiple comparisons.

The administration of carrageenan in the intraplantar region of mice induced a significant edema formation over time (Figure 4A,B), in which the edematogenic peak was obtained in the fourth hour after challenge. A single oral treatment with COEFC at 8.35, 41.75 and 83.5 mg/kg significantly reduced the edema by 83.1%, 81.5%, and 79.4%, respectively, between 60 and 240 min after challenge, in comparison with the control group, which occurred at all time points: 8.35 mg/kg dose (T60: 73.1%, T120: 75.0%, T180: 88.6%, and T240: 89.5%), 41.75 mg/kg (T60: 65.9%, T120: 60.7%, T180: 90.0%, and T240: 97.4%), and 83.5 mg/kg (T60: 63.4%, T120: 62.5%, T180: 82.9%, and T240: 97.4%).

The administration of dextran (1%) promoted a significant edema formation at all time intervals. The maximal edematogenic effect was recorded 2h after challenge but decreased from the fourth hour (Figure 4C,D). A single oral treatment with the COEFC at 8.35, 41.75, and 83.5 mg/kg doses resulted in a significant decrease in edema by 80.3%, 80.6%, and 85.9%, respectively, when compared to the control group. When correlating this action with the time points and intervals, the COEFC reduced the edema at all times (60–240 min), at all tested doses: 8.35 mg/kg (T60: 74.7%, T120: 80.2%, T180: 77.4%, and T240: 91.2%), 41.75 mg/kg (T60: 75.9%, T120: 76.0%, T180: 81.3%, and T240: 91.2%), and 83.5 mg/kg (T60: 72.3%, T120: 82.3%, T180: 92.5%, and T240: 98.5%). Because no significant differences between the tested doses were observed, the effect of COEFC was not dose dependent (Figure 4C,D). It has previously been reported that both OEFC and 1,8-cineole had anti-edematogenic effects that were not dose dependent [29]. Here, comparable outcomes were obtained in the carrageenan-induced edema when the complex was administered at lower doses.

Promethazine (6 mg/kg, p.o.), a histamine-receptor antagonist, manifested a significant anti-edematogenic effect of 73.8% in comparison with the control group. In the time intervals between the first and fourth hour, the drug was always effective (T60: 69.9%, T120: 72.9%, T180: 69.9%, and T240: 85.3%). The COEFC presented an anti-edematogenic activity at all tested doses, with the greatest inhibition observed at the fourth hour after challenge (Figure 4C,D).

In a previous study conducted by Martins et al. (2017) [29], the essential oil from Croton rhamnifolioides (OEFC) at doses of 25, 50, and 100 mg/kg/v.o. promoted a reduction of 71.9%, 78.1%, and 71.9%, while 1,8-cineole at doses of 10.3, 20.6, and 41.3 mg/kg/v.o. showed a reduction of 80.5%, 79.9%, and 79.2%, respectively, in the formation of edema when compared to the control group. Corroborating with this study, the complex (COEFC) had the advantage of achieving a similar and significant effect, reducing edema at lower doses.

Although edema inhibition was observed at all analyzed time points, it was not dose dependent. As expected, indomethacin (10 mg/kg) significantly reduced edema by 83.1% when compared to the control group, demonstrating an anti-inflammatory action at all time intervals (T60: 73.1%, T120: 69.6%, T180: 92.9%, and T240: 100%) (Figure 4A,B).

### 3.5. Paw Edema Induced by an Intraplantar Administration of Histamine or Arachidonic Acid

**Figure 5:**
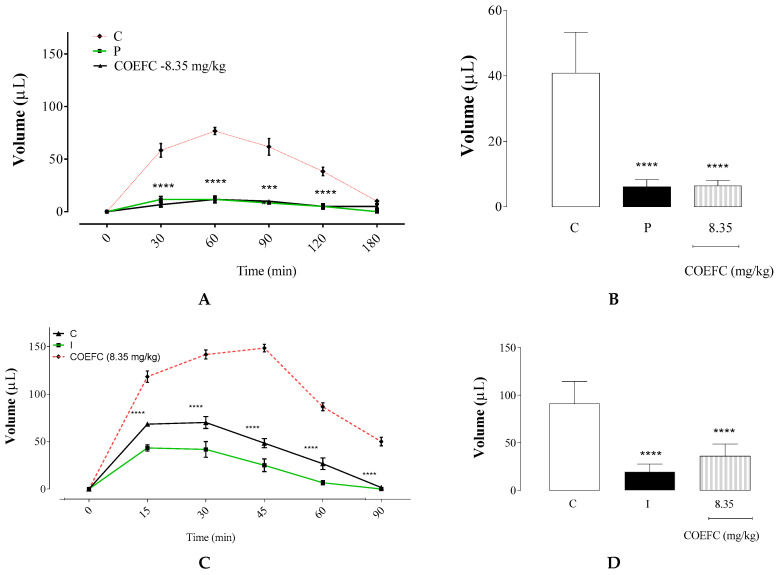
Effect of the COEFC (8.35 mg/kg/p.o.) on paw edema (**A**,**B**)intraplantar injection of 1% histamine and (**C**,**D**) induced by the 1% arachidonic acid in mice. (**A**) Edema value induced by histamine (μL) up to 180 min between control (C) (saline/tween—0.1 mL/10 g/p.o.), promethazine (P) (6 mg/kg/p.o.), (**B**) Results of median paw volume (μL) the 3rd hour after the moment of application, (**C**) Edema value induced by arachidonic acid (μL) up to 90 min between the control (C) (saline/tween—0.1 mL/10 g/p.o.), indomethacin (I) (10 mg/kg/s.c.) and COEFC (8.35 mg/kg/p.o.) groups. (**D**) Results of median paw volume (μL) 90 min after the moment of application. Values represent the mean ± S.P.M. (*n* = 6/group). **** *p* < 0.0001 vs. control; Statistical analysis: ANOVA followed by the Dunnett’s test with multiple comparisons.

**Figure 6:**
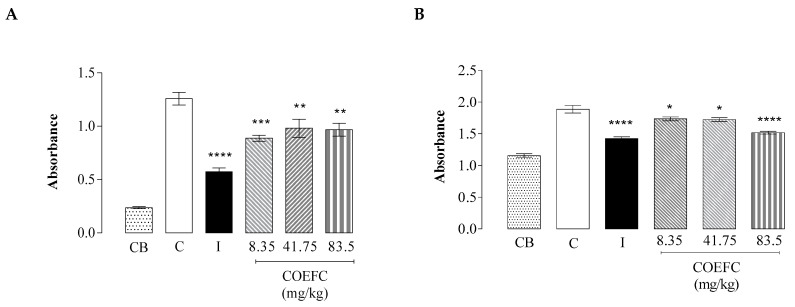
Effect of the COEFC (8.35, 41.75, and 83.5 mg/kg/p.o.) relative to vascular permeability by Evans Blue extravasation (**A**) and quantification of the total proteins (g/dL) (**B**). Vascular permeability by Evans Blue extravasation between control (C) (saline—0.1 mL/10 g/p.o.), baseline (CB) (saline—0.1 mL/10 g/p.o.), indomethacin (I) (10 mg/kg/s.c.), and COEFC (8.35, 41.75 and 83.5 mg/kg/p.o.). Values represent the mean ± S.P.M. (*n* = 6/group). * *p* < 0.05; ** *p* < 0.01; *** *p* < 0.001; **** *p* < 0.0001 vs. control; Statistical analysis: ANOVA followed by the Dunnett’s test with multiple comparisons.

Figure 5A shows that the histamine-induced edema reached an edematogenic peak at 60 min (T60) after challenge. However, it declined following 120 min (T120). The COEFC (8.35 mg/kg/p.o.) and promethazine (6 mg/kg/p.o.) had anti-edematogenic effects of 84.4% and 85.0%, respectively, at 8.35 mg/kg, when compared with the control group, demonstrating that there were no significant differences between these treatments (Figure 6B). The COEFC showed an effect at all analyzed times (T30: 88.6%, T60: 84.8%, T90: 83.8%, T120: 87.0%, and T180: 50%), with the highest percent inhibition value at 30 min (T30) after histamine challenge and the administration of the antagonist promethazine showed action at all time intervals (T30: 80.0%, T60: 84.8%, T90: 86.5%, T120: 87.0%, and T180: 100%). However, these treatments obtained maximal efficacy at 180 min after edema induction with histamine. According to Martins et al. (2017) [29], a single treatment with the OEFC (25 mg/kg/p.o.) or 1,8-cineole (10.83 mg/kg) demonstrated anti-edematogenic effects, decreasing the edema by 74.1% and 66.0%, respectively; thus, we can conclude that, comparatively, in the present study, COEFC presented even better results, and the complexation process with cyclodextrin demonstrates an advantage to promote more bioavailable to pharmacological.

The administration of arachidonic acid induced a paw edema that reached a peak at 45 min (T45) and declined from 60 min (T60) (Figure 5C). The COEFC (8.35 mg/kg) and indomethacin (10 mg/kg) significantly reduced this edema by 60.6% and 78.6%, respectively, when compared to the control group. Thus, although both treatments were effective at all time intervals (COEFC (T15: 50.7%, T30: 50.6%, T45: 67.4%, T60: 69.2%, and T120: 96.7%) and indomethacin: (T15: 63.4%, T30: 70.6%, T45: 83.1%, T60: 92.3%, and T120: 100%), they had greater activity after 60 min of induction. Moreover, the action indomethacin was directly proportional to the time after induction (Figure 5D). At the same conditions, both OEFC (25 mg/kg/p.o.) and 1,8-cineole (10.3 mg/kg) significantly reduced the edema by 64.4% and 50.8%, as previously reported [29].

### 3.6. Vascular Permeability by Evans Blue Extravasation

An evaluation of the vascular permeability demonstrated that the COEFC at the 8.35, 41.75, and 83.5 (mg/kg) inhibited this parameter by 29.46%, 22.10%, and 23.16%, respectively, compared to the control group. These data corroborate with those observed in the analysis of protein extravasation, in which the same concentrations caused inhibitions of 8.0%, 8.5%, and 19.6%, respectively, suggesting an efficient reduction of the inflammatory exudate. In both assays (Figure 6A,B), no dose-dependent effects were observed, since no significant differences between the tested doses were seen. The control drug indomethacin significantly reduced vascular permeability by 54.5% and protein extravasation by 24.5%, demonstrating the effectiveness of a non-steroidal anti-inflammatory drug (NSAID) in inhibiting these parameters. In an earlier study by Martins et al. (2017) [29], the OEFC (25 mg/kg/p.o.) and 1,8-cineole (10.3 mg/kg/p.o.) reduced both vascular permeability (31.0% and 31.5%, respectively) and the total proteins (10.9% and 16.8%, respectively), as demonstrated in the present study, using the COEFC at lower doses. These findings corroborate with the evidence that these treatments may affect the action of inflammatory mediators involved in edema formation.

### 3.7. Granuloma Induced by Cotton Pellet Implantation

**Figure 7:**
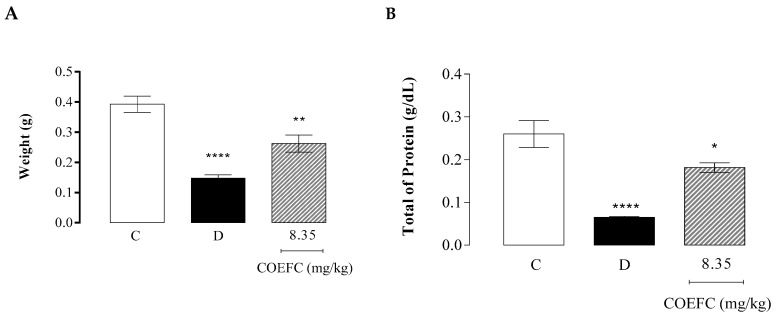
Effect of the COEFC (8.35 mg/kg/p.o.) on granuloma induced by the implantation of cotton pellets; (**A**) Mass (g) of the pellets and (**B**) total protein (g/dL). Mass of the cotton pellets dry (A) and quantification of total proteins (B) between the control (C) mL/10 g/vol), dexamethasone (D) (5 mg/kg/p.o.), and COEFC (8.35 mg/kg/p.o). Values represent the mean ± S.P.M. (*n* = 6/group). * *p* < 0.05; ** *p* < 0.01; **** *p* < 0.0001 vs. control; Statistical analysis: ANOVA followed by the Dunnett’s test with multiple comparisons.

The COEFC (8.35 mg/kg) and dexamethasone (5 mg/kg) significantly decreased the dry mass of the pellets by 33.16% and 62.50%, respectively, when compared to the control group (Figure 7A). These data corroborate with the total protein content present in the granuloma, since treatment with the COEFC (8.35 mg/kg) and dexamethasone reduced the total proteins by 30.4% and 76.9%, respectively (Figure 7B). Accordingly, in the study by Martins et al. (2017) [29], the OEFC (25 mg/kg/p.o.) and 1,8-cineole (10.3 mg/kg/p.o.) caused significant reductions in the weight of the granulomatous tissue (46.7% and 20.0%, respectively) and the protein content of the granuloma (38.9% and 85.1%) in comparison with the control group. These results corroborate with those obtained in the paw edema and vascular permeability tests.

### 3.8. Molecular Docking Analysis

Virtual-screening docking provides a suitable indication of the possible biological activities of compounds, reducing the cost and time of drug discovery studies. It also estimates the binding strength and the energy of the complex in addition to calculating the binding affinity using scoring functions. This study used molecular docking analysis to understand ligand–protein interactions between COX-2 and 1,8-cineole and other therapeutically relevant compounds. The six compounds successfully docked into the COX-2 active site, with binding energies in the range of −4.8 to −10.4 kcal/mol, as shown in Table 2. However, a lower interaction energy was obtained for celecoxib with −10.4 kcal/mol or 70.80nMol equivalence. The inhibition constant and the binding energy indirectly measure the tendency to form an enzyme–ligand complex, and therefore, represent the probability that a molecule binds to a given enzyme, which in turn is directly related to the pharmacological potency of this molecule. The results of the docking showed that the selective COX-2 inhibitor celecoxib presented the highest inhibition constant (Ki) = 70.8 nM, while meloxicam (moderately selective COX-2 inhibitor) presented Ki = 3.02 µM and diclofenac and naproxen (non-selective COX-1/COX-2 inhibitors had Ki of 13.2 and 14.26 µM, respectively, corroborating with the pharmacological potency of NSAIDs (Table 2).

All ligands tested in the coupling experiments showed binding to the active site of COX-2, which may be explained in terms of π–π, hydrophobic, and polar interactions, in addition to halogen and hydrogen interactions, among others. A docking analysis with prostaglandin E_2_ (PGE_2_) showed that the end carboxylate group has a proximal interaction with the PGH_2_ Arg 120, Tyr 355, Tyr 385, and Ser 530 chains. The interactions with the prostaglandin endoperoxide synthases and ring present in PGE_2_ are stabilized by van der Waals interactions with Phe381, Leu384, Tyr385, and Trp387. Interactions may also occur with the Trp387, Tyr348, and Tyr385 in the hydrophobic cavity of the COX-2 binding site and a lipophilic region defined by Leu93, Val116, and Leu359. The 1,8-cineole can assume a variety of closely related conformations stabilized by several hydrophobic interactions in the upper part of the site with residues, such as Leu352, Phe381, Tyr385, Trp387, Phe515, Val523, and Ala527. However, Tyr385 and Ser530 contribute to enforcing the hydrophobic interactions. The common pocket is occupied by groups that are similar in size, such as the phenyl group in the case of ibuprofen.

The inhibition constant, Ki, is an indication of how potent an inhibitor is and represents the inhibitor concentration at which 50% of inhibition is observed. A drug may have high affinity to a receptor and bind maximally even at relatively low concentrations. Thus, as 1,8-cineole showed better affinity (31.12 versus 433.11µM) than prostaglandin E_2_, this natural product could dislocate prostaglandin E_2_ from the pocket site.

**Figure 8:**
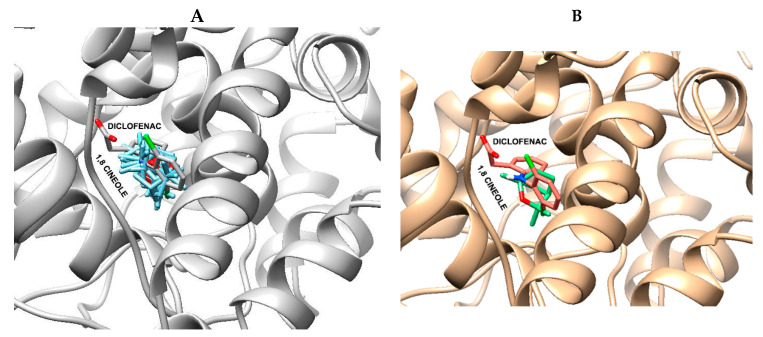
Docking of 1,8 cineole in the active site of COX-2 enzyme: (**A**) all conformations (blue stick) and diclofenac (gray stick) and (**B**) best binding conformations (green) and diclofenac (pink stick). Hydrogen atoms of the amino acid residues have been removed to improve clarity.

Figure 8A shows the orientation of 1,8-cineole in 10 conformations of the best binding energy, as well as the best binding conformation of 1,8 cineole into the COX-2 active site. However, Figure 8B shows the best binding conformations (green) compared with the diclofenac (pink stick) binding pose. These docking results may help in the understanding of the anti-inflammatory action of *Croton rhamnifolioides* essential oil through the molecular interactions of 1,8-cineole with COX-2 enzyme. These results corroborate the data obtained in vivo, especially in the tests of paw edema induced by carrageenan or arachidonic acid, which are dependent on COX-2-mediated prostaglandin synthesis.

## 4. Discussion

This study evaluated the formation of inclusion complexes of the *Croton rhamnifolioides* essential oil with β-CD. Our data demonstrated that the most effective complexation was obtained using the co-evaporation method, which was proven through analysis by DSC, DTG, Karl Fisher, and MeV. Cyclodextrins (CD) represent one of the complexing agents most commonly used in the pharmaceutical industry due to their ability to improve the physical, chemical, and biological properties of bioactive molecules, especially those extracted from plants [58], although there is controversy regarding side effects in the renal system, in which orally administered β-CDs have been shown to induce limited toxicity. Jiang et al. (2017) and Gould and Scott (2005) [59,60] evaluated the inclusion of triterpene acids in β-CD and obtained favorable results regarding the fit and binding to the β-CD cavity [59,60]. Their data demonstrated that the molecular interaction and complex formation between these compounds is given by the increased number of hydrogen bonds and hydrophobic interactions that promote conformational adjustments to take maximum advantage of the weak van der Waals forces in the hydrophobic cyclodextrin cavity [61]. The physicochemical analysis by DSG/DTG, showing the presence of an endothermic peak, suggests the dehydration of the β-CD cavity [62]. Thus, the absence of an endothermic peak from the inclusion complex by co-evaporation reinforces an efficient complexation between the OEFC and β-CD. The study by Sherje A. P. et al. (2017) [63] on the characterization and formulation of etodolac (a NSAID) inclusion complexes showed that the complexes prepared by the co-evaporation method had higher drug solubility compared to the spray drying and physical mixing methods.

Sithole et al. (2017) [6] evaluated the integrity of drugs in inclusion complexes and concluded an entrapment of 85% of a drug in the hydrophobic inner core of the complex does not promote loss of the substance in question and maintains its integrity, ensuring the clinical effect of the drug and only modifying the solubility patterns to the improve bioavailability. Additionally, monoterpenes are generally easily complexed by β-CD due to the size of their cavity and internal lipophilic pore affinity, which accommodates simple compounds, such as those formed by two isoprene units, especially monoterpenoid ether, such as 1,8-cineole [41]. As for the *Croton* genus, Aguiar et al. (2014) [7] describes the formation of the inclusion complex between the *C. zenhtneri* essential oil (OEC) and β-CD using the co-precipitation method in which infrared spectroscopy (IR spectroscopy) and DSC analyses verify the greater stability of the OEC complex compared to the free OEC, justified by the introduction of the oil into the β-CD cavity [7].

The reduction in the water content, as observed in the Karl Fisher analysis, reinforces the complexation of the OEFC with the β-CD cavity in all methods. This event may be associated with the replacement of water molecules in the β-CD cavity by the studied drug molecules [55]. The Karl Fisher method is considered an important tool in the determination of the water content and water binding with the surface of CDs, since, according to Marreto et al. (2008) [64], desolvation is important for the quality of the molecular encapsulation of hydrophobic substances through the substitution of water molecules. A recent review by de Lima et al. (2012) [65] listed several studies showing that inclusion complexes with essential oils, especially with β-CD, were obtained despite the low solubility of the oils [65]. In fact, this method remains the best option in virtue of the low cost and easy production, guaranteeing great bioavailability in the biological environment [39].

Many of us use essential oils as natural remedies or consume them in teas. However, publications on the toxicity of these products are heterogenous. They include allergic reactions, acute toxicity, irritation, corrosiveness, sensitization, phototoxicity, carcinogenicity, reprotoxicity, and teratogenicity. These effects have been reported for various essential oils, especially from chronic or repeated exposure. The essential oil obtained from Wormwood, which contains a high concentration of thujone, is potentially fatal [66]. Its ingestion can cause seizures, kidney failure, stomach cramps, paralysis, nightmares, vomiting, muscle breakdown, and other distasteful symptoms [67]. *Artemisia annua*, a plant used in the treatment of malaria, is an important source of artemisinin, in addition to having an essential oil rich in 1,8-cineole [66]. *Myristica fragrans* is a familiar product in our spice cabinets, adding a special touch to cookies, cakes, and pies. However, it contains 1,8-cineole (eucalyptol) that can be dangerous when ingested in excess, causing vomiting, drowsiness, and eventually a coma [68,69]. Furthermore, *Eucalyptus* oil, rich in 1,8 cineole, causes vomiting and diarrhea in addition to other side effects [25,70]. Previous studies have shown that eugenol, a constituent of the essential oil of many species, such as *Thymus capitatus*, *Thymus cilicus*, and *Thymus vulgaris*, is hepatotoxic and may cause fatal hepatic failure [71]. The toxicity of some essential oils is a limiting factor in their therapeutic use. Thus, the incorporation of these products into inclusion complexes may represent an important alternative in therapy, since these compounds can increase the bioavailability and reduce the toxicity and consequently the side effects.

The anti-edematogenic and anti-inflammatory effects of the essential oil obtained from the leaves of *Croton rhamnifolioides* (OEFC) have been previously established. Martins and colleagues demonstrated that both the OEFC (25 mg/kg) and 1,8-cineole (10.3 mg/kg) efficiently reduced the inflammatory response both in the acute and chronic phases [29]. Thus, in this new study, we demonstrated that complexation in cyclodextrins caused an increment in the bioavailability of these products, since similar pharmacological outcomes were obtained with the systemic administration of 1/3 of the effective dose (8.35 mg/kg). This reduction in the effective dose may represent a reduction in the risk of the toxic effects that limit the medicinal use of the essential oils [72].

On the other hand, the results obtained from the topical administration of the β-CD inclusion complex indicate that this treatment do not present significant anti-inflammatory effects in ear edema induced by croton oil. This possibly occurs due to a reduced capacity of penetration in the epidermal epithelial membranes [73]. According to Anjana et al. (2013) [74], the chemical structure, molecular weight, and low partition coefficient in the ethanol/water of CDs contribute to their permeability in water, hindering the diffusion process through biological membranes [74]. Natural CDs, such as β-CD, have limited water solubility when compared to synthetic CDs. However, their ability to easily complex with monoterpenes and their known use in oral formulations in more than 35 products in the market make β-CDs the most widely used CDs in the pharmaceutical [40,41,75].

Regarding the kinetics of cyclodextrin complexation, two different phenomena may occur, affecting the velocity of the process: A complexation with the outer face, which occurs with slower speed due to the difficulty of desolvatization of the water; and complexation in the inner face that presents a more lipophilic characteristic favoring a faster complexation [39]. However, the permeability of the complex through the skin is low due to its hydrophilic character. Therefore, although the results for the topical test showed a trend of anti-inflammatory action, they were not significant (*p* < 0.05). This tendency can also be explained by the permeability of the components of the complexed essential oil on the outer face of the cyclodextrin, which may undergo absorption. However, the anti-inflammatory effect of OEFC and 1,8-cineole topically can be justified by the lipossibility property, which facilitates its penetration through the skin, characteristically present in in terpenoid derivatives. In the present study, the COEFC showed a systemic anti-inflammatory effect in paw edema models induced by carrageenan and dextran at all doses tested, which corroborated the research done by Martins et al. (2017) on the inflammatory effects of OEFC and 1,8-cineole [29]. 

These findings suggest that the complex effectively released constituents capable of interfering in the production of chemical mediators, such as vasoactive amines and prostaglandins, responsible for vasodilation and the formation of inflammatory exudates [76]. A recent study by Martins et al. (2017) showed that the systemic administration of same *C. rhamnifolioides* essential oil at doses of 25, 50, 100, and 200 mg/kg resulted in a significant reduction of carrageenan and dextran-induced edema [29]. However, the complexation of this oil maintained effective responses even with the administration of lower doses (8.35, 41.75, and 83.5 mg/kg), providing a reduction of 33.4% in the lowest tested, which suggests an improvement in the bioavailability of the OEFC in the complex β-CD. These data are supported by previous studies that demonstrated that analgesic and anti-inflammatory drugs complexed with β-CD present increased bioavailability and efficacy [5]. 

In the histamine-induced paw edema test, OEFC and 1,8-cineole [29] and COEFC significantly reduced edema, suggesting an interference in this pathway through a possible reduction in the release by mast cells [77,78]. Histamine is a chemical preformed mediator that is found in cellular vesicles, primarily in mast cells and macrophages. In inflammatory responses, this mediator is particularly involved in the increase in vascular permeability, which is mediated by G protein-coupled histamine receptors [79].

The COEFC showed an anti-inflammatory effect by significantly reducing arachidonic acid-induced paw edema, which corroborates the study conducted by Martins et al. (2017) in validating the anti-inflammatory action of OEFC and 1,8-cineole [29]. In this model, the edema is a result of the action of mediators produced by the oxidation of this fatty acid by enzymes, such as cyclooxygenase and lipoxygenase, and by free radicals, such as hydrogen peroxide. The chemical products of this oxidation, including prostaglandins and leukotrienes, are responsible for increased permeability, migration, and leukocyte activation [80,81]. Therefore, it is suggested that the COEFC interfered with the oxidative processes involved in arachidonic acid metabolism.

COX-2 expression is dramatically regulated during inflammation, and as such it is a potential target to explain, at least in part, the anti-inflammatory action of the essential oil and 1,8 cineole. This hypothesis is supported by molecular docking studies and corroborates the findings of Martins et al. (2017) and Beer et al. (2017), indicating that 1,8-cineole is a possible COX-2 inhibitor [29,82].

The results presented in this study are comparable to those described previously, in which all structural details of the binding of NSAIDs to cyclooxygenase show that hydrogen bonds and hydrophobic interactions represent major contributions to the complex formation. Hydrogen bonds have been demonstrated to be important for COX-2 inhibitory ligands, especially in the binding of indomethacin and naproxen to the cyclooxygenase active site above Arg-120 and the side chain of Tyr-355 [83]. Diclofenac forms H-bonds through its carboxylate with the Tyr-385 and Ser-530 residues of this enzyme [84]. Meloxicam forms hydrogen bonds with Ser-530 as well as with two coordinated water molecules complexed to Tyr-385/Ser-530 and Arg-120/Tyr-355. Additionally, the selectivity of this drug for COX-2 is resultant from the subtle fit of the structure in the neighborhood of the Phe-518 residue that interferes with secondary shell residues Ile-434 for COX-1 and Val-434 for COX-2 [85]. Diarylheterocycle COX-2 inhibitors, such as celecoxib, rofecoxib, and nimesulide, bind to the cyclooxygenase active site above Arg-120 and insert sulfonamide or sulfone groups into a side pocket bordered by Val-523 [86]. In addition, a polar nitro group can form hydrogen bounds with Ser-530 and/or Tyr-385, and the sulfone may bind in the side pocket, corroborating the results seen in the present study, which showed similar interactions [87,88].

The hydrophobic interactions are also important for stabilization of the COX-2-ligand complex, involving the amino acid residues that surround the active site: Leu384, Tyr385, Trp-387, Phe518, Met522, Val-523, and Ser530 [89]. However, other residues, such as Met-113, Val-116, Leu-117, Ile-345, Val-349, Leu-531, Leu-534, Met-535, Ile523, Ala526, Gly526, Glu524, Ser353, Leu359, Val349, and Leu352, present hydrophobic interactions with the phenyl ring present in different ligands [85,90]. Although 1,8-cineol does not exhibit hydrogen bonds, it assumes conformations that are stabilized by diverse hydrophobic interactions with residues like those described above.

Corroborating with the effects of the essential oil on histamine and eicosanoids pathways, vascular permeability was determined through the Evans Blue assay, in which the dye has affinity for plasma albumin, resulting in the formation of a dye–albumin complex that can be quantified and used to express protein extravasation through the endothelial barrier in inflammatory processes [52]. The results presented in this study demonstrated a significant reduction in vascular permeability and total proteins in the peritoneal fluid of mice treated with COEFC. These data corroborate with the research done by Martins and collaborators (2017) that affirms the antiedematogenic action of OEFC and 1,8-cineole, indicating that the anti-edematogenic effects obtained in the paw edema models induced by dextran, histamine, and arachidonic acid may be associated with an action of the complex on vascular permeability [29].

Again, the effective dose of the COEFC was lower than for the isolated oil or 1,8-cineole. These data are corroborated by a study by Rodrigues et al., (2017), in which complexation with β-CD improved the anti-inflammatory effects of an essential oil obtained from *Ocimum basilicum* (OEOB/β-CD [37].

The maintenance of the inflammatory process induced by persistent pathogens, foreign bodies, and immune alterations triggers a chronic inflammatory response mediated mainly by cytokines, such as tumor necrosis factor (TNF-α) and transforming growth factor (TGF-β), responsible for the recruitment of fibroblasts leading to the formation of chronic granulomas [91]. In the model of chronic inflammation induced by cotton pellets, OEFC, 1,8-cineole [29], and COEFC significantly reduced the mass of the cotton pellets and the total protein content. This result also correlates with the acute inflammatory models demonstrated in this study. Other studies have demonstrated similar results validating the observed anti-inflammatory effect [29,92,93]

Additionally, 1,8-cineole is known for its effect on modulating the NF-κB (nuclear factor kappa B) pathway, which regulates multiple aspects of both innate and adaptive immune functions, serving as a fundamental mediator of inflammatory responses [94]. In addition, the NF-κB transcriptional factor induces the expression of several proinflammatory genes, including those encoding cytokines and chemokines, as well as participating in inflammasome regulation [95]. In addition, other constituents present in the OEFC and COEFC have anti-inflammatory activities already reported, including spathulenol [30], caryophyllene oxide [31], trans-caryophyllene [32], β-elemene [33], α-terpineol [34], tterpinen-4-ol [35], and α-Phellandrene [36]. Therefore, they may contribute to the anti-inflammatory profile of these samples.

Recent studies showed that the pharmacological activities of essential oils obtained from species, such as *Ocimum basilicum* [93,96], *Lippia grata* [97], and *Hyptis martiusii* [98], as well as the activity of isolated monoterpenes, such as (-)-linalool [99] and carvacrol [100], are optimized when complexed with CDs. In some cases, complexation may improve the bioavailability without affecting the pharmacological effect of the transported molecule. However, for essential oils and terpenes, it has been observed that complexation optimizes the pharmacological effects, reducing the effective doses [91,101,102]. In this study, it was found that at smaller doses, the anti-inflammatory effects of the COEFC were better or similar to those of the free OEFC or 1,8-cineole [29]. Therefore, complexation could reduce the potential toxicity of the essential oil, especially when used in repeated doses in the treatment of chronic diseases, such as arthritis.

## 5. Conclusions

The physicochemical characterization of the *Croton rhamnifolioides* essential oil showed that co-evaporation (CE) is the best inclusion method for β-cyclodextrin complexation due to a higher stability. In comparison with the free OEFC and 1,8-cineole, the complexed oil (COEFC) showed better anti-inflammatory activity in mice models of acute and chronic inflammation, which may be related to a possible increase in bioavailability, and therefore, an improvement of the pharmacological effects, thus reducing the effective dose.

The results obtained in the present study confirm the traditional use of *Croton rhamnifolioides* for the treatment of inflammatory conditions and suggest that its action may originate from an interference of histamine- and arachidonic acid-dependent pathways. In addition, the molecular docking data indicate a favorable binding of the oil with the COX-2 enzyme. Taken together, these data confirm both the anti-inflammatory potential of OEFC and the applicability of the complex as a drug delivery system. 

Therefore, because complexation with cyclodextrin improved the pharmacological effects of the OEFC, reducing the dose required to maintain the anti-inflammatory effects in comparison with previous results, we believe that this complexation may also contribute to a reduced incidence of toxic effects.

## Figures and Tables

**Table 1:** Mass loss percentages obtained by thermogravimetry/derivative thermogravimetry and Karl Fisher titration from the OEFC (*Croton rhamnifolioides* essential oil), β-CD (β cyclodextrin), MF(physical mixture), MA (malaxage) and CE (co-evaporation) samples.

Sample	1st Step/% 30–220 °C	2nd Step/% 220–270 °C	3rd Step/% 270–380 °C	4th Step/% 380–500 °C	% H_2_O
**OEFC**	97.2 ± 1.48	1.62 ± 0.35	0.88 ± 0.10	0.52 ± 0.01	0.91 ± 0.06
**β-CD**	12.8 ± 1.34	0.1 ± 0.07	76.6 ± 1.48	3.7 ± 0.14	13.75 ± 0.39
**MF**	19.4 ± 0.07	0.5 ± 0.01	70.9 ± 1.13	2.7 ± 0.98	13.45 ± 0.78
**MA**	10.1 ± 0.2	2.4 ± 0.01	92.2 ± 2.92	3.2 ± 0.84	11.27 ± 0.32
**CE**	8.5 ± 1.69	2.6 ± 0.28	83.4 ± 1.48	3.2 ± 0.56	12.80 ± 0.27

**Table 2:** Binding energy of the interaction the different ligands with prostaglandin G/H synthase 2 by the autodock procedure (cyclooxigenase 2).

Ligand	Energy (kcal/mol)	Est. Inhibition Constant, Ki* (µM or ηM)
Prostaglandin E2 (Natural ligand)	−4.8	433.11 µM
Celecoxib	−10.4	70.80 ηM
Diclofenac	−8.4	13.2 µM
Meloxicam	−9.1	3.02 µM
Naproxen	−8.4	14.26 µM
1,8-Cineole	−6.0	31.12 µM

* Theorical Inhibition Constant calculated by autodock function. Ki unit is µM = micromoles or ηM = nanomols.
